# Regularized regression in ultra-small chemometric datasets: A methodological case study using FTIR spectra of Schiff bases

**DOI:** 10.1371/journal.pone.0341850

**Published:** 2026-06-01

**Authors:** Khudhayr Abdullah Rashedi, Tariq Saleh Alshammari, Khalid Mohammed Alshammari, Talal Abdulrahman Alanazi, Javid Shabbir, Tahir Mehmood

**Affiliations:** 1 Department of Mathematics, College of Science, University of Ha’il, Hail, Saudi Arabia; 2 Department of Statistics, University of Wah, Wah Cantt, Pakistan; 3 School of Natural Sciences, National University of Sciences and Technology, Islamabad, Pakistan; University of Lucknow, INDIA

## Abstract

This study is not intended to establish a predictive framework for reaction yield. Instead, it is framed as a methodological investigation examining the statistical behavior and instability of regularized regression techniques when applied to ultra-small, high-dimensional chemometric datasets. The analysis is based on a curated dataset of Schiff base compounds (*n* = 21) for which post-synthesis Fourier Transform Infrared (FTIR) spectra and experimentally reported reaction yields are available. Structural information for all compounds is fully disclosed to ensure chemical transparency. Descriptive physicochemical properties, including molecular weight, physical appearance, retention factor (Rf), melting point, and reaction yield, are summarized to characterize the dataset; however, only yield (%) is used as the response variable in the subsequent statistical analyses. Baseline-corrected and normalized FTIR spectra were transformed into a high-dimensional explanatory matrix and analyzed using regularized regression approaches designed for high collinearity and p≫n settings, specifically sparse Partial Least Squares (sPLS) and Elastic Net regression. Model behavior was examined using leave-one-out cross-validation (LOOCV), which is more appropriate for extremely small datasets where conventional train–test splitting is unreliable. Given the severe sample-size limitation, the analysis is interpreted as a methodological illustration rather than a generalizable predictive framework. Model outputs are therefore discussed primarily in terms of coefficient sparsity, variability, and stability under regularization rather than predictive accuracy. Overall, the study demonstrates the practical challenges and statistical instability that arise when regression-based machine learning techniques are applied to ultra-small spectral datasets. The results highlight the importance of cautious interpretation and methodological transparency when chemometric models are developed under severe sample-size constraints.

## 1 Introduction

Schiff bases, formed through the condensation of primary amines with aldehydes or ketones, constitute an important class of compounds in organic and coordination chemistry due to their structural versatility and wide-ranging applications in catalysis, materials science, and biological systems [1–3]. Although their synthesis is typically straightforward, reported reaction yields often vary substantially depending on substituent effects, molecular structure, and experimental conditions. Understanding such variability remains challenging, particularly in small experimental studies where detailed reaction-condition information may be incomplete.

Recent developments in data-driven chemistry have demonstrated that statistical and machine learning approaches can assist in identifying empirical patterns in chemical datasets [4,5]. However, many studies in this area rely on large and diverse datasets containing extensive reaction descriptors, which limits their applicability to small laboratory-scale datasets commonly encountered in exploratory synthetic studies. Under such circumstances, chemometric analysis may still provide descriptive insight, provided that methodological limitations—particularly those related to sample size and model stability—are explicitly acknowledged.

Fourier Transform Infrared (FTIR) spectroscopy is routinely used for the structural characterization of Schiff bases because it captures vibrational features associated with functional groups such as imines, hydroxyl groups, and aromatic systems [6]. FTIR spectra represent high-dimensional molecular signatures that reflect structural characteristics of the synthesized compounds. However, these spectra are collected after synthesis and therefore cannot be interpreted as mechanistic determinants of reaction yield. Instead, they can be viewed as descriptive molecular fingerprints that may reflect structural similarities among compounds.

The high dimensionality and strong collinearity of FTIR spectral variables present significant challenges for conventional regression modeling, particularly when the number of observations is small relative to the number of predictors [7]. In such (p≫n) settings, regression models can become unstable, highly sensitive to small perturbations in the data, and prone to overfitting. Regularization techniques have therefore been widely adopted in chemometrics to control model complexity and stabilize estimation in high-dimensional datasets.

Two approaches are particularly relevant in this context. Sparse Partial Least Squares (sPLS) combines latent-variable projection with variable selection, enabling dimensionality reduction while retaining interpretable spectral features [8,9]. Elastic Net regression integrates $\ell_{1}$ and $\ell_{2}$ penalties, allowing stable coefficient estimation in the presence of strongly correlated predictors [10,11]. Both methods are widely used in chemometric modeling, yet their statistical behavior under extremely small sample conditions has received comparatively limited attention.

In this study, we analyze a curated dataset consisting of 21 synthesized Schiff base compounds for which FTIR spectra and experimentally reported yields are available. Structural information for all compounds is fully disclosed to ensure chemical transparency. Descriptive physicochemical properties—including molecular weight, physical appearance, retention factor (Rf), melting point, and yield—are summarized to characterize the dataset.

The primary aim of this work is not to construct a predictive model for reaction yield nor to infer mechanistic relationships between spectral measurements and reaction outcomes. Instead, the study is framed as a methodological case study designed to examine the statistical behavior of regularized regression techniques when applied to ultra-small, high-dimensional chemometric datasets.

Because the dataset contains only twenty-one observations accompanied by a large number of spectral predictors, it represents a typical (p≫n) scenario. Under these conditions, regression models are susceptible to instability, overfitting, and strong dependence on validation procedures. By applying regularized regression methods and evaluating their behavior using leave-one-out cross-validation (LOOCV), the present study aims to illustrate the methodological challenges associated with modeling spectral data under extreme sample-size constraints.

The results are therefore interpreted cautiously as descriptive methodological observations rather than as evidence of predictive capability or chemical causality. In this sense, the contribution of the study lies in highlighting the statistical limitations and interpretational challenges that arise when machine learning methods are applied to ultra-small chemometric datasets.

## 2 Methodology

This section describes the data structure, preprocessing procedures, and modeling strategy used for the exploratory analysis of Schiff base yields. Given the limited sample size, particular emphasis is placed on transparency, regularization, and cautious validation to minimize overfitting and optimistic bias.

### 2.1 Structural description of synthesized Schiff bases

**Fig 1:**
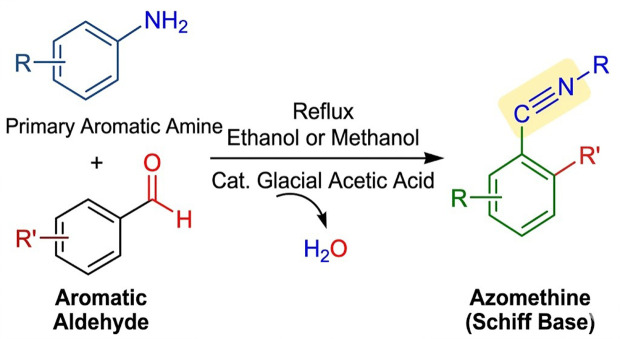
General synthetic pathway for Schiff base formation via condensation of aromatic aldehydes with primary aromatic amines producing azomethine (-CH = N-) derivatives.

**Fig 2:**
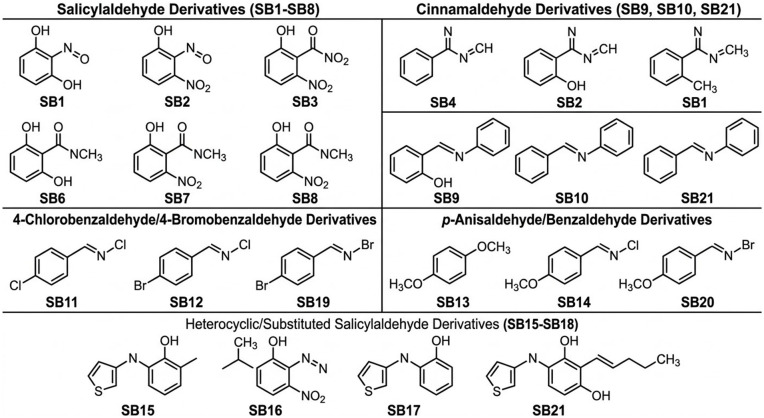
Two-dimensional chemical structures of the twenty-one synthesized Schiff bases used in the chemometric modeling analysis.

Twenty-one Schiff bases were synthesized through condensation reactions between substituted aromatic aldehydes and primary aromatic amines under reflux conditions in ethanol or methanol with catalytic amounts of glacial acetic acid, see the Figs 1 and 2, and Table 1. The reaction produces azomethine derivatives characterized by the formation of the imine functional group (-CH = N-).

**Table 1:** Synthesized Schiff bases with corresponding aldehyde and amine precursors.

ID	Aldehyde	Amine	IUPAC Name
SB1	Salicylaldehyde	p-Anisidine	2-((4-methoxyphenylimino)methyl)phenol
SB2	Salicylaldehyde	4-Aminophenol	2-((4-hydroxyphenylimino)methyl)phenol
SB3	Salicylaldehyde	4-Nitro-1,2-phenylenediamine	2-((2-amino-5-nitrophenylimino)methyl)phenol
SB4	Salicylaldehyde	1,4-Phenylenediamine	2-((4-aminophenylimino)methyl)phenol
SB5	Salicylaldehyde	2,4,6-Trimethylaniline	2-((2,4,6-trimethylphenylimino)methyl)phenol
SB6	Salicylaldehyde	o-Toluidine	2-((o-tolylimino)methyl)phenol
SB7	Salicylaldehyde	Aniline	2-(phenylimino)methylphenol
SB8	Salicylaldehyde	4-Amino-2-nitrophenol	2-((4-hydroxy-2-nitrophenylimino)methyl)phenol
SB9	Cinnamaldehyde	p-Anisidine	(E)-3-phenyl-N-(4-methoxyphenyl)prop-2-en-1-imine
SB10	Cinnamaldehyde	4-Amino-2-nitrophenol	(E)-3-phenyl-N-(4-hydroxy-2-nitrophenyl)prop-2-en-1-imine
SB11	4-Chlorobenzaldehyde	Aniline	N-(4-chlorobenzylidene)aniline
SB12	4-Bromobenzaldehyde	4-Amino-2-nitrophenol	4-bromobenzylidene-4-amino-2-nitrophenol
SB13	p-Anisaldehyde	4-Amino-2-nitrophenol	4-methoxybenzylidene-4-amino-2-nitrophenol
SB14	Benzaldehyde	4-Amino-2-nitrophenol	4-(benzylideneamino)-2-nitrophenol
SB15	5-Nitro-2-thiophenecarboxaldehyde	4-Amino-2-nitrophenol	2-nitro-4-(((5-nitrothiophen-2-yl)methylene)amino)phenol
SB16	3,5-Dichlorosalicylaldehyde	4-Amino-2-nitrophenol	2-nitro-4-(((3,5-dichloro-2-hydroxyphenyl)methylene)amino)phenol
SB17	5-Nitro-2-thiophenecarboxaldehyde	4-Aminophenol	4-(((5-nitrothiophen-2-yl)methylene)amino)phenol
SB18	3,5-Dichlorosalicylaldehyde	4-Aminophenol	2,4-dichloro-6-(((4-hydroxyphenyl)imino)methyl)phenol
SB19	4-Bromobenzaldehyde	p-Anisidine	4-bromobenzylidene-4-methoxyaniline
SB20	Benzaldehyde	p-Anisidine	N-benzylidene-4-methoxyaniline
SB21	Cinnamaldehyde	Aniline	(E)-3-phenyl-N-phenylprop-2-en-1-imine

The aldehydes used in the synthesis include salicylaldehyde, 3,5-dichlorosalicylaldehyde, 4-chlorobenzaldehyde, trans-cinnamaldehyde, 5-nitro-2-thiophenecarboxaldehyde, p-anisaldehyde, benzaldehyde, and 4-bromobenzaldehyde. The amine precursors include p-anisidine, aniline, o-toluidine, 2,4,6-trimethylaniline, 4-aminophenol, 4-amino-2-nitrophenol, 4-nitro-1,2-phenylenediamine, and 1,4-phenylenediamine.

Structural diversity among the synthesized compounds arises from variations in substituent groups including hydroxyl (-OH), nitro ($-{\text{NO}}_{2}$), chloro (-Cl), methyl ($-{\text{CH}}_{3}$), and methoxy ($-{\text{OCH}}_{3}$) groups located on the aromatic rings. The complete list of synthesized Schiff bases including IUPAC names and SMILES representations is provided in S1 Table.

### 2.2 General reaction scheme

### 2.3 Chemical structures of synthesized compounds

For descriptive purposes, physicochemical characteristics including molecular weight, physical appearance, retention factor (Rf), melting point, and experimentally reported yield (%) are summarized to provide an overview of the chemical space under investigation. These variables are used solely for dataset characterization and are not jointly modeled as predictors in the machine learning framework.

Post-synthesis Fourier Transform Infrared (FTIR) spectra were recorded for each compound over the range of 500–4000 cm^−1^. The response variable for all machine learning analyses is the synthetic yield (%), treated as a continuous outcome.

### 2.4 Spectral preprocessing

Raw FTIR spectra were preprocessed to reduce instrumental and baseline-related artifacts prior to modeling. First, baseline correction was applied to remove low-frequency background drift. Subsequently, Savitzky–Golay smoothing was used to attenuate high-frequency noise while preserving peak structure. Finally, spectra were normalized to ensure comparability across samples.

All preprocessing steps were applied identically to all spectra. During cross-validation, preprocessing parameters were estimated exclusively from training data and subsequently applied to the held-out sample to prevent information leakage.

### 2.5 Construction of the explanatory matrix

After preprocessing, FTIR absorbance values at each wavenumber were assembled into a high-dimensional explanatory matrix **X**. Each row corresponds to a compound and each column represents a spectral variable. Given the dimensionality of the spectral data relative to the small sample size, no unregularized regression models were considered.

The analysis is explicitly exploratory, and FTIR variables are interpreted as empirical proxies for molecular structure rather than mechanistic determinants of yield.

### 2.6 Regression models

Two regression approaches were evaluated due to their ability to handle multicollinearity and high-dimensional predictors.

#### 2.6.1 Sparse partial least squares (sPLS).

Sparse Partial Least Squares regression was employed to jointly perform dimension reduction and variable selection. By introducing sparsity constraints on the loading vectors, sPLS reduces model complexity while retaining interpretable latent components. The number of latent components and the number of variables retained per component were treated as tunable parameters.

#### 2.6.2 Elastic net regression.

Elastic Net regression combines $\ell_{1}$ (Lasso) and $\ell_{2}$ (Ridge) penalties, making it suitable for correlated spectral variables. This approach encourages sparse yet stable coefficient estimates and is particularly appropriate for small-sample, high-dimensional settings. Two hyperparameters were considered: the mixing parameter α∈[0,1] and the regularization strength λ>0.

### 2.7 Model validation and hyperparameter tuning

Model performance was assessed using Leave-One-Out Cross-Validation (LOOCV). For Elastic Net and sPLS, internal cross-validation within the training set was used to select optimal hyperparameters.

This nested validation strategy was adopted to reduce the risk of information leakage and overly optimistic performance estimates. Model performance was summarized using the root mean square error (RMSE) on test data. Model sparsity, quantified by the number of selected spectral variables, was used as a secondary criterion to assess interpretability.

### 2.8 Statistical limitations under extreme sample constraints

The dataset analyzed in this study contains only twenty-one observations, which places fundamental limits on the statistical reliability of any predictive modeling exercise. When the number of predictors greatly exceeds the number of observations (p≫n), regression models become highly sensitive to small perturbations in the data and may exhibit substantial variance in estimated parameters.

Although regularization techniques such as sPLS and Elastic Net can mitigate overfitting by shrinking coefficients and selecting variables, they cannot compensate for fundamental limitations imposed by extremely small sample sizes. Consequently, model performance estimates obtained through cross-validation should not be interpreted as reliable indicators of predictive capability.

Instead, the modeling framework adopted in this study is intended primarily to illustrate methodological behavior under extreme small-sample conditions. The results therefore serve as descriptive observations regarding model stability and variable selection rather than as evidence of generalizable predictive relationships.

### 2.9 Reproducibility and implementation

All analyses were performed in the R statistical environment (version 4.5.1). The mixOmics package was used for sparse Partial Least Squares modeling, and the glmnet package was used for Elastic Net regression. All random procedures were executed using fixed seeds to ensure reproducibility.

Given the small dataset size, results are interpreted as exploratory and hypothesis-generating rather than confirmatory.

## 3 Results and discussion

This section presents the exploratory results obtained from applying regularized regression models to post-synthesis FTIR spectral data for Schiff base compounds. Given the limited sample size, the analysis focuses on comparative model behavior, stability, and interpretability rather than definitive prediction or mechanistic inference.

**Fig 3:**
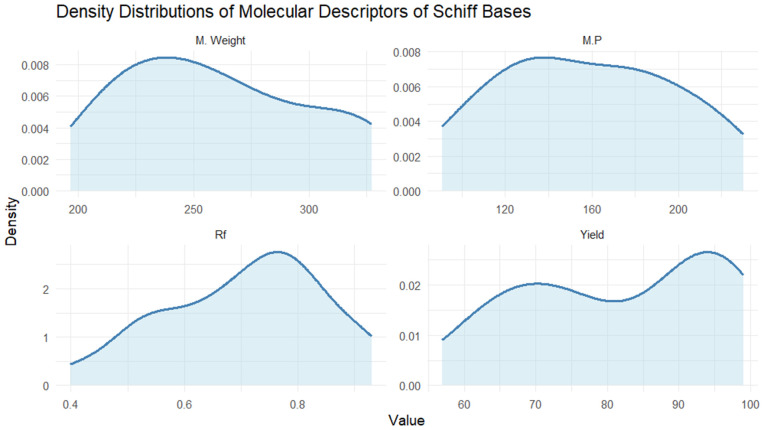
The density plots representing the distributions of molecular weight, Rf value, melting point, and yield.

**Fig 4:**
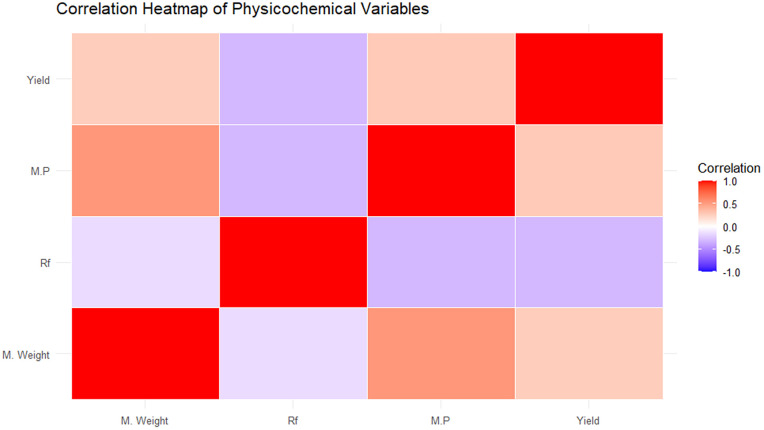
It shows a correlation heatmap displaying pairwise relationships between molecular descriptors of Schiff bases.

**Fig 5:**
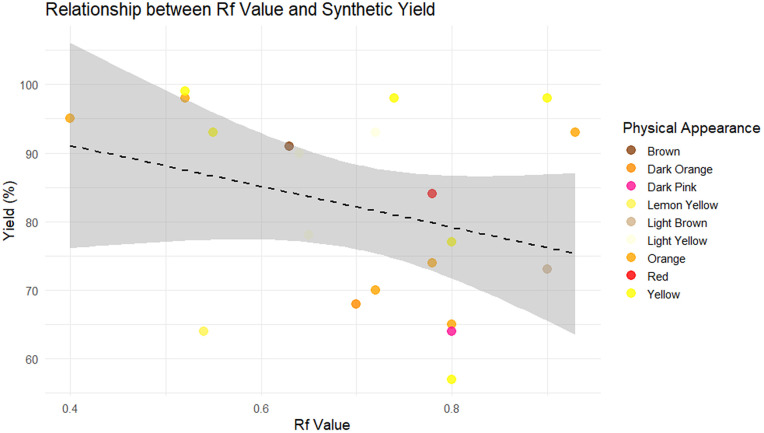
The relationship between Rf values and synthetic yield across different physical appearances.

**Fig 6:**
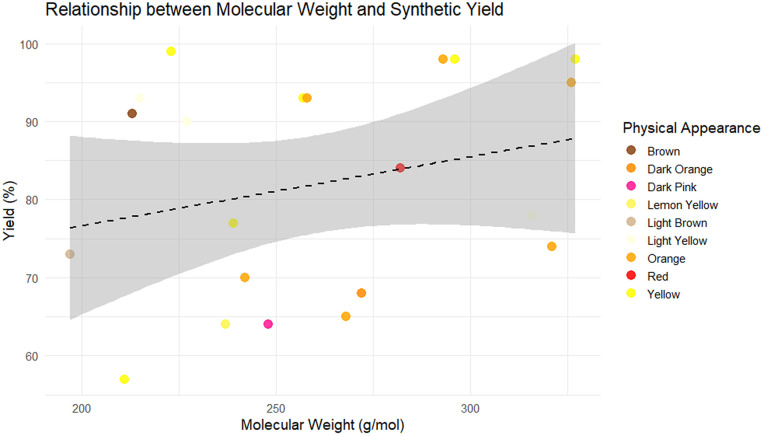
The relationship between molecular weight and synthetic yield with color-coded physical appearances.

Descriptive statistics of the dataset were first examined to characterize the chemical space under investigation. Molecular weight, physical appearance, retention factor (Rf), melting point, and yield exhibited moderate variability across the 21 Schiff base compounds, indicating a heterogeneous but limited dataset, see Figs 3–6. These descriptors are reported solely for contextual understanding and were not used jointly as predictors in the machine learning models. Yield (%) was treated as the sole response variable in all regression analyses.

**Fig 7:**
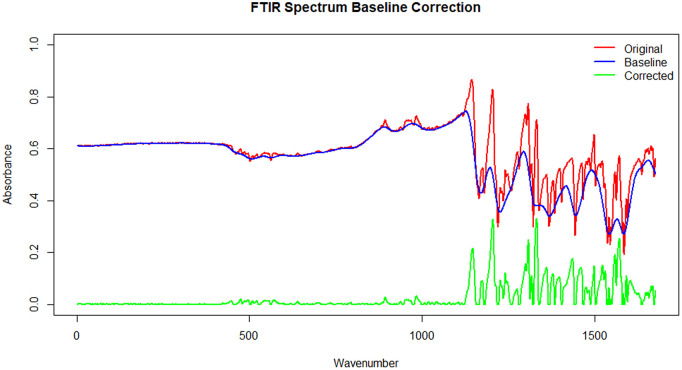
FTIR baseline correction illustrating the original spectrum (red), the estimated baseline (blue), and the corrected spectrum (green).

Baseline correction and smoothing of FTIR spectra resulted in the effective removal of background drift and high-frequency noise, producing cleaner and more comparable spectral profiles across compounds, see Fig 7. The corrected spectra preserved characteristic absorption bands associated with imine (C = N), hydroxyl (O–H), and aromatic functional groups, ensuring that subsequent modeling was based on consistent spectral representations. No chemical interpretation is inferred at this stage; preprocessing is intended strictly to improve numerical stability and comparability.

**Fig 8:**
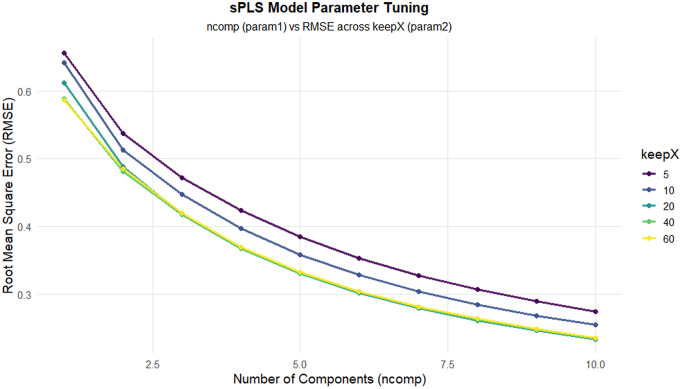
It shows the sPLS tuning surface, where RMSE varies with the number of components (*n*_*comp*_) and the variable selection parameter (*keepX*).

**Fig 9:**
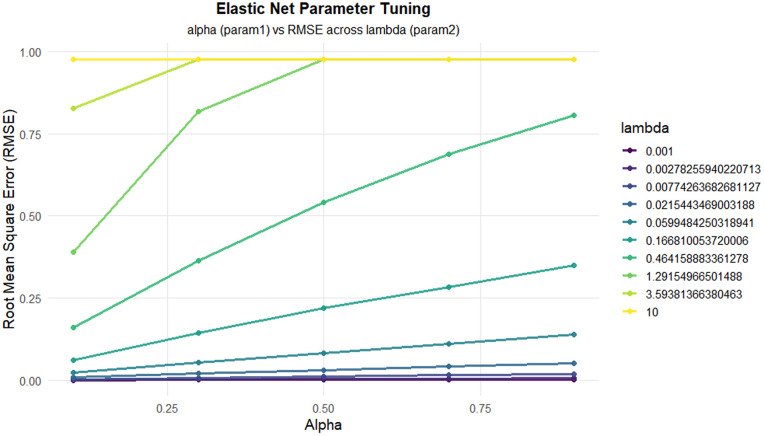
It presents Elastic Net tuning, illustrating RMSE variation across α and λ values.

**Fig 10:**
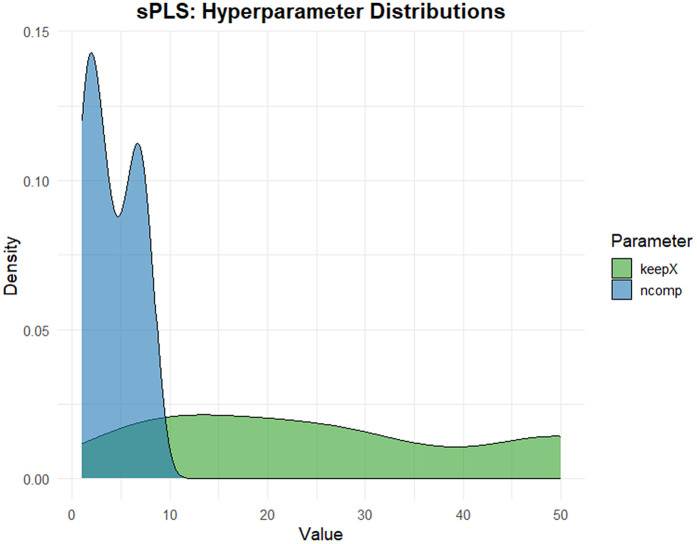
The distribution of sPLS hyperparameters (*n*_*comp*_ and *keepX*) across repeated runs.

**Fig 11:**
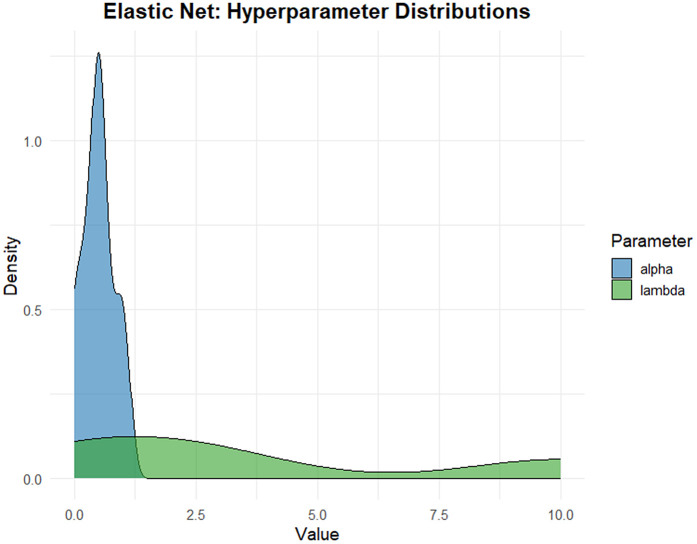
The distribution of Elastic Net hyperparameters (α and λ).

The two regression approaches—sparse Partial Least Squares (sPLS) and Elastic Net—were optimized using Leave-One-Out Cross-Validation (LOOCV) with strict separation between training and testing data, see Figs 8 and 9. Hyperparameter distributions across resampling iterations revealed differences in model stability, see Figs 10 and 11.

**Fig 12:**
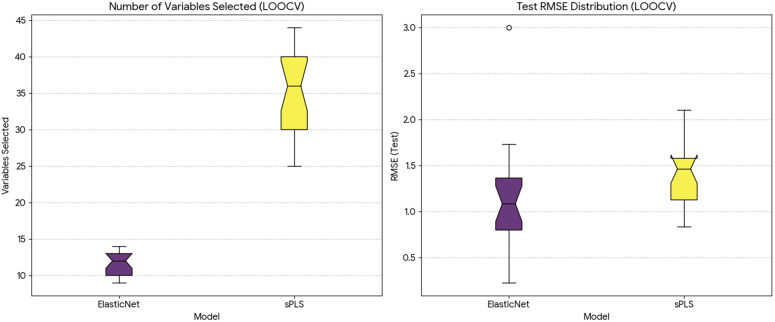
Boxplots summarizing model performance and sparsity across Leave-One-Out Cross-Validation (LOOCV). The upper panel shows the distribution of RMSE values on held-out test observations for sPLS and Elastic Net models. The lower panel presents the number of selected spectral variables across LOOCV iterations.

Elastic Net exhibited relatively narrow variability in the selected regularization parameters, indicating stable convergence under different train–test splits. In contrast, sPLS showed moderate variability in the number of retained components and selected variables, reflecting sensitivity to data partitioning while still enforcing sparsity through its regularization mechanism, see Fig 12. These observations highlight the importance of regularization and model simplicity when analyzing high-dimensional spectral data with limited observations.

Model performance was evaluated using root mean square error (RMSE) on held-out test observations across LOOCV iterations. Overall, Elastic Net achieved slightly lower median RMSE values and exhibited smaller variability across validation runs, whereas sPLS demonstrated greater dispersion in error estimates. Differences in RMSE between the two models were not statistically significant, and no claims of model superiority are made based on formal hypothesis testing.

Given the small dataset size, performance metrics should be interpreted cautiously. The observed differences primarily reflect model robustness to data perturbation rather than generalizable predictive accuracy. Elastic Net demonstrated the most consistent balance between error magnitude and variability, supporting its suitability for exploratory modeling under constrained sample conditions.

**Fig 13:**
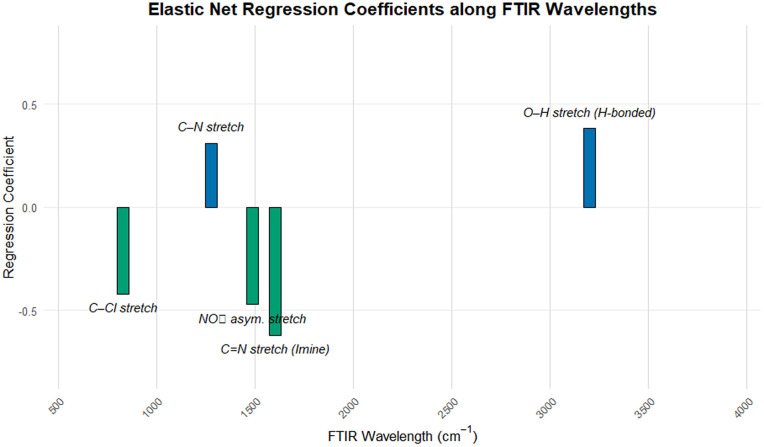
Elastic Net regression coefficients mapped along the FTIR wavenumber axis, highlighting spectral regions selected by the regularized model.

Model interpretability was assessed through the number of retained spectral variables. Both Elastic Net and sPLS consistently produced sparse solutions, selecting a limited subset of FTIR wavenumbers across iterations, see Fig 13. This behavior enhances interpretability by reducing the influence of redundant and highly correlated spectral variables and by highlighting spectral regions that contribute most strongly to the statistical model.

These associations should be interpreted strictly as statistical correlations rather than mechanistic determinants of synthetic yield. Given the high dimensionality of the spectral data and the limited sample size, it is not possible to disentangle true chemical effects from correlated structural features or experimental artifacts. Nonetheless, the consistency of selected regions across regularized models suggests that FTIR spectra capture reproducible structural information reflecting similarities among the synthesized compounds.

From a methodological perspective, the analysis illustrates how regularized regression techniques can be applied cautiously to high-dimensional spectral data even when the available sample size is extremely limited. Elastic Net provided comparatively stable performance and consistent sparsity, while sPLS offered additional dimensionality reduction through latent components but showed greater sensitivity to tuning parameters. These observations reinforce the importance of regularization and careful validation when exploratory chemometric analyses are performed on small datasets.

### 3.1 Statistical limitations under extreme sample constraints

The dataset used in this study consists of only twenty-one observations (*n* = 21), which represents an extremely small sample size relative to the dimensionality of the FTIR spectral predictors. Such conditions correspond to a high-dimensional setting where the number of explanatory variables substantially exceeds the number of observations (p≫n). Under these circumstances, regression-based modeling becomes statistically unstable and highly sensitive to small variations in the data.

We fully acknowledge that no statistical or machine learning technique can compensate for fundamental limitations in sample size. Regularization methods such as sparse Partial Least Squares (sPLS) and Elastic Net can help control coefficient magnitude and reduce variance, but they do not eliminate the inherent uncertainty associated with ultra-small datasets. Consequently, the models evaluated in this study should not be interpreted as reliable predictive tools.

To address this limitation, the study has been explicitly reframed as a methodological case study intended to examine the behavior of regularized regression approaches under extreme sample constraints rather than to establish a predictive framework for reaction yield. Model results are therefore interpreted descriptively and cautiously, emphasizing patterns of model stability, sparsity, and variability rather than predictive accuracy or generalizability.

These limitations highlight an important methodological point for chemometric applications: when spectral dimensionality is high and the number of observations is extremely limited, model outputs should be interpreted primarily as exploratory statistical summaries. Robust predictive modeling and mechanistic interpretation would require substantially larger datasets with independent validation.

## 4 Conclusion

This study serves primarily as a methodological illustration of the statistical challenges that arise when applying regression-based machine learning techniques to extremely small chemometric datasets. Using a dataset of twenty-one synthesized Schiff base compounds with high-dimensional FTIR spectral measurements, the analysis examined the behavior of regularized regression approaches under a typical (p≫n) setting.

Among the evaluated models, regularized linear methods demonstrated comparatively stable behavior in the presence of strong multicollinearity and limited observations. Elastic Net regression provided consistent coefficient shrinkage and sparse solutions across validation runs, while sparse Partial Least Squares (sPLS) enabled dimensionality reduction through latent components and facilitated exploratory feature selection. These approaches illustrate how regularization can help stabilize model estimation when conventional regression techniques become unreliable in high-dimensional spectral settings.

Importantly, the spectral regions identified by the models should be interpreted strictly as statistical associations rather than mechanistic determinants of synthetic yield. Because FTIR measurements were obtained after synthesis and the dataset size is extremely limited, the results cannot support causal interpretation or reliable predictive modeling. Instead, the findings should be viewed as descriptive observations regarding model stability, sparsity, and variability in ultra-small chemometric datasets.

Overall, this work highlights both the utility and the limitations of applying regularized chemometric models to small FTIR datasets in organic synthesis. Future studies incorporating substantially larger datasets, independent validation samples, and additional reaction descriptors would be necessary to support more robust statistical analysis and deeper chemical interpretation.

## Supporting information

S1 Table The complete list of synthesized Schiff bases including IUPAC names and SMILES representations. (CSV)
